# Genome-wide methylation analysis of a large population sample shows neurological pathways involvement in chronic widespread musculoskeletal pain

**DOI:** 10.1097/j.pain.0000000000000880

**Published:** 2017-02-15

**Authors:** Gregory Livshits, Ida Malkin, Maxim B. Freidin, Yudong Xia, Fei Gao, Jun Wang, Timothy D. Spector, Alex MacGregor, Jordana T. Bell, Frances M.K. Williams

**Affiliations:** a Department of Twin Research and Genetic Epidemiology, King's College London, London, United Kingdom; b Department of Anatomy and Anthropology, Sackler Faculty of Medicine, Tel Aviv University, Tel Aviv, Israel; c Beiging Genomic Institute-Shenzhen, Shenzhen, China; d School of Medicine, University of East Anglia, Norwich, United Kingdom

**Keywords:** DNA methylation, EWAS, Chronic widespread pain, Twin, Epigenome, MeDIPseq

## Abstract

Chronic widespread musculoskeletal pain (CWP), has a considerable heritable component, which remains to be explained. Epigenetic factors may contribute to and account for some of the heritability estimate. We analysed epigenome-wide methylation using MeDIPseq in whole blood DNA from 1708 monozygotic and dizygotic Caucasian twins having CWP prevalence of 19.9%. Longitudinally stable methylation bins (lsBINs), were established by testing repeated measurements conducted ≥3 years apart, n = 292. DNA methylation variation at lsBINs was tested for association with CWP in a discovery set of 50 monozygotic twin pairs discordant for CWP, and in an independent dataset (n = 1608 twins), and the results from the 2 samples were combined using Fisher method. Functional interpretation of the most associated signals was based on functional genomic annotations, gene ontology, and pathway analyses. Of 723,029 signals identified as lsBINs, 26,399 lsBINs demonstrated the same direction of association in both discovery and replication datasets at nominal significance (*P* ≤ 0.05). In the combined analysis across 1708 individuals, whereas no lsBINs showed genome-wide significance (*P* < 10-8), 24 signals reached p≤9E-5, and these included association signals mapping in or near to *IL17A*, *ADIPOR2,* and *TNFRSF13B*. Bioinformatics analyses of the associated methylation bins showed enrichment for neurological pathways in CWP. We estimate that the variance explained by epigenetic factors in CWP is 6%. This, the largest study to date of DNA methylation in CWP, points towards epigenetic modification of neurological pathways in CWP and provides proof of principle of this method in teasing apart the complex risk factors for CWP.

## Abstract 2

Supplemental Digital Content is Available in the Text.

We analysed epigenome-wide methylation in blood DNA in Caucasian female twins. Bioinformatics analyses of the associated methylation signals showed enrichment for neurological pathways in CWP.

## 1. Introduction

Chronic widespread pain (CWP) is a common musculoskeletal condition with a lifetime prevalence of 5% to 15% in European populations.^44,52^ The estimates of prevalence vary depending on definition; however, it is agreed that the frequency of CWP condition increases sharply in females and with increasing age^41,55,62^ and that CWP is very costly to society.^23^ The etiology of CWP is complex and poorly understood, but CWP has been shown by several groups to be heritable with heritability estimates usually exceeding 30%.^34,48^ Of the many risk factors that have been proposed for CWP, increased body mass index (BMI) is one of the strongest and most consistently reported.^36,40,57^ Our work has shown the influence of BMI on CWP risk to be through increased fat mass.^33^ We have also shown that the risk of CWP correlates inversely with the circulating levels of biochemical factors related to androsterone metabolism, in particular epiandrosterone sulphate and dihydroepiandrosterone sulphate.^8,33^

Epidemiological studies of CWP show that it co-occurs with other chronic pain and affective syndromes such as irritable bowel syndrome, anxiety, and depression. Our studies and those of others suggest that shared genetic factors (pleiotropy) may affect simultaneously different chronic pain syndromes.^8,58^ Over the last decade, several candidate genes have been identified that are potentially linked to CWP^31,45,47^, however, only a few of these genes have been reliably replicated. Agnostic approaches such as at genome-wide linkage and association have produced some novel findings^13,16^ but together they explain only a fraction of the genetic contribution.

It is well established that genes undergo substantial changes in their pattern of expression throughout the course of life. These patterns are, to an extent, coordinated by epigenetic mechanisms such as DNA methylation that mainly occurs at cytosine residues in the CpG dinucleotides within gene promoter regions.^4^ There is evidence that DNA methylation contributes to the development of many diseases, eg, cancer^46^ and cardiovascular disease^18^ through interfering with gene expression. Epigenetic processes could plausibly be involved in the pathogenesis of chronic pain.^3,29^ For instance, histone acetylation and methylation, additional important mechanisms of regulation of gene expression, have been implicated in the etiology of chronic pain^29^ and neuropathic pain in particular.^21^ However, experimental data on this topic remain sparse and we are aware of only 1, extremely limited clinical (10 patients), epigenome-wide association study (EWAS).^37^ Our group has previously conducted an EWAS of the heat pain sensitivity in small sample of 100 volunteer twins (free of CWP), and detected epigenetic change in both novel and established candidate pain genes.^5^ We set out in the current study, therefore, to analyse genome-wide changes in blood DNA methylation levels in a much larger, population sample characterised for CWP.

## 2. Methods

### 2.1. Study sample

The subjects in the present study were from the TwinsUK Adult Twin Registry, described in detail elsewhere.^39^ The registry had been collected from the general population through national media campaigns in the United Kingdom and without ascertainment for any particular characteristics, diseases, or traits. The sample included 1708 women (age ranged from 17 to 82 years, with average 51.8 ± 13.7 years) with questionnaire responses to CWP assessment (see below) and having methylation level measurements (n = 2002, some individuals were repeatedly measured). The sample comprised 565 monozygotic (MZ) twin pairs, 244 dizygotic (DZ) twin pairs, and 90 singletons (individual twins with missing cotwin data). For all singletons, the CWP status of their respective sibling was known. All participants gave written informed consent before entering the study and the St. Thomas' Hospital Research Ethics Committee had approved the project.

### 2.2. CWP definition

The London Fibromyalgia Epidemiology Study Screening Questionnaire had been sent to twins for self-completion, without reference to the cotwin.^61^ Twins with pain on both left and right sides of the body, above and below the diaphragm, duration of 7 days or more within the preceding 3 months were considered as cases. These twins had participated in the CWP genome-wide association study meta-analysis^47^ and in the recent omics study.^33^ In clinical visits, body height and weight were measured and BMI (in kg/m^2^) was calculated.

### 2.3. Smoking scores

The present sample included 1307 individuals, for whom the information on smoking habits was available. Of these, 588 (45%) individuals were ever smokers including 99 current smokers and 489 ex-smokers.

### 2.4. Blood cell composition

Whole blood cell (WBC) subtype counts were obtained for 441 individuals using flow cytometry analysis of peripheral blood.^43^ WBC subtype cell counts were available for 4 cell types: neutrophils, eosinophils, monocytes, and lymphocytes.

### 2.5. MeDIP-sequencing and DNA methylation quantification

This methodology was described by us recently elsewhere (Livshits et al).^32^ Briefly, whole blood DNA was fragmented to a smear of 200 to 500 bp with the Bioruptor NGS System (Diagenode) and subsequently methylated DNA was immunoprecipitated using the Magnetic Methylated DNA Immunoprecipitation Kit (Diagenode).^5,28^ After efficiency and sensitivity assessment by qPCR, MeDIP-seq libraries were prepared by amplification, purification, and validation followed by high-throughput sequencing (Illumina HiSeq2000) that generated ∼50 million 50 bp single-end reads. After adapter and base quality trimming, sequencing reads were mapped to hg19 using BWA v0.5.9.^23^ Alignments with low quality scores (Q < 10) and duplicates were filtered, which resulted in an average of 15,684,723 uniquely mapped reads that were subsequently extended to 350 bp to represent the average MeDIP fragment size. Fragments per kilobase per million were quantified in bins (methylation sites) of 500 bp (250 bp overlap) genome wide using MEDIPS v1.6.^10^

### 2.6. Design of the study and statistical analysis

**Figure 1.**
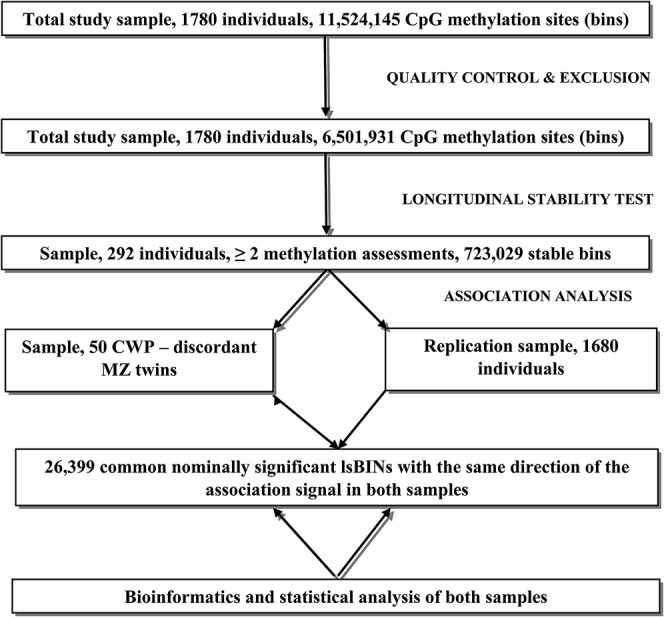
The main lines of the design of the present study.

The methylation levels were assessed at 11,524,145 CpG sites, genome wide (bins) in each of the 1708 individuals in the sample. The data analysis was carried out in several stages, diagrammatically shown in Figure 1. Individuals having repeated measures of methylation (n = 388) included 292 samples analysed in the same laboratory batch, which were used to identify longitudinally stable DNA methylation regions. After quality control and exclusion of all bins for which ≥ 20% individuals had zero methylation, 6,501,931 bins remained. To test longitudinal stability of the methylation levels, we computed Pearson correlations between these bins in all individuals whose methylation levels were measured 2 times at least 3 years apart (mean = 7.0 years, SD = 1.2 years, range: 3.4-10.8 years). Bins displaying significant intraindividual correlation (*P* < 0.05) were considered as longitudinally stable (called “lsBIN” below) and were used for subsequent analysis. To clarify the extent to which the interindividual variation in methylation levels of longitudinally stable methylation bins (lsBINs) was governed by additive genetic factors we computed correlations between the twin measurements for each of the lsBINs for MZ and DZ pairs separately.

Next, we identified bins associated with CWP occurrence using a discovery set of 50 pairs of MZ-twins discordant (affected/nonaffected) for CWP. Here, the difference between the date of DNA extraction and CWP assessment was < 5 years for 46 pairs and 5 to 10 years for 4 MZ twin pairs. Paired Student's *t* test was performed to compare methylation levels in CWP-affected and nonaffected twins.

The nominally significantly (*P* < 0.05) associated lsBINs were then examined in the replication sample (total remaining sample, N = 1608 individuals) implementing *t* test after adjustment of methylation levels for age and age^2^ (as the relationship with age was nonlinear). The *P*-values for the results obtained in 2 nonoverlapping samples were combined using Fisher method.^14^ Once the most significant lsBINs have been identified, the association of each bin with CWP was tested using modified multiple logistic regression. In addition to methylation levels, we also examined the effect of BMI, smoking, and WBC subtype counts, simultaneously adjusting for age and cotwin CWP-affection status (covariates).

To evaluate the relative contribution of additive genetic factors (narrow sense heritability, h^2^) and methylation in top-ranked bins to CWP, we carried out modified variance decomposition analysis based on a classical polygenic concept of the heritability of threshold traits.^15^ The analysis was conducted using Mendelian Analysis Package (MAN) for family-based samples.^34,35^

### 2.7. Multiple testing correction

The false discovery rate for multiple testing under dependency was estimated.^7^ Methylation levels in neighbouring and other genomic sites may not be independent, so we calculated the effective number of independent tests using eigenvalues of the correlation matrix of tested bins after a modified version of Li and Ji (2005).^22^ This procedure was carried out in the total sample (n = 1708) on lsBINs. Significance was considered if the combined results of the discovery and replication sets reached epigenome-wide significance (*P* ≤ 10E-7) estimated from the number of independent tests.

### 2.8. Functional genomic annotations and gene ontology analysis

The top lsBINs displaying significant association with CWP in the discovery set and validated in the replication sample were mapped to genomic regions implementing the HapMap repository (https://hapmap.ncbi.nlm.nih.gov/) and GeneCards database (http://www.genecards.org/).

Gene ontology (GO) analysis was carried out based on both sets of results. First, we assigned the lsBINs to nearby ENSEMBL genes using *MEDIPS* package for R.^30^ For genes with multiple bins assigned we retained the lsBIN with the lowest *P*-value for association with CWP. Using Fisher approach we took the combined *P*-values and carried out GO analysis using the *weight01* algorithm implemented in the *topGO* package for R.^1^ The statistical significance of over-representation of GO terms was estimated using Fisher exact test. Two GO domains have been analysed, Biological Process (BP) and Cellular Component (CC). QIAGEN's Ingenuity Pathway Analysis (IPA; QIAGEN Redwood City, www.qiagen.com/ingenuity) was used for pathway analysis.

## 3. Results

### 3.1. Descriptive statistics and identification of longitudinally stable DNA methylation regions

The basic descriptive statistics of the study sample, by affection status are given in Table 1. The CWP-affected females were older, had higher BMI and tended to smoke significantly more than unaffected individuals. Note, however, the smoking data was available for a subset (n = 1307) participants.

**Table 1:**
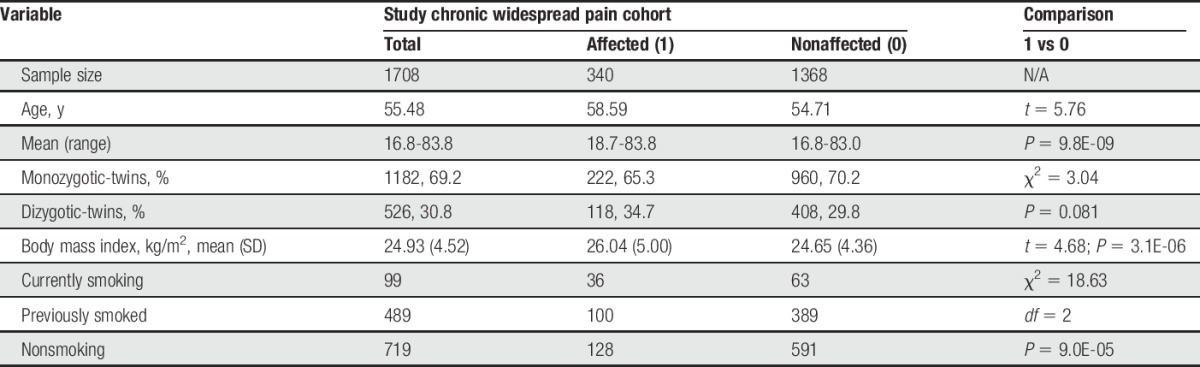
Basic descriptive statistics of the study sample.

A total of 6,501,931 bins (56.4%) remained (from the initial 11,524,145 bins) after quality control testing and exclusion of bins displaying zero methylation levels in >20% of the study sample. Nominally significant (*P* < 0.05) Pearson correlations between the repeated measurements (R_repeat_) in 292 selected individuals were found for 723,029 bins (lsBINs; 6.3% of the initial 11,524,145 bins). The size of the correlations varied between 0.114 and 0.905, with the median 0.145.

### 3.2. Heritability of lsBIN methylation levels

The analysis of methylation levels per bin within the twin pairs showed that MZ twin correlations (R_MZ_s) had mean 0.210 ± 0.016; whereas DZ twins correlations (R_DZ_s) had mean 0.161 ± 0.018. Both R_MZ_ and R_DZ_ estimates clearly increased with R_repeat_, with the corresponding linear correlations lying between 0.516 (for R_MZ_) and 0.347 (for R_DZ_) (*P* < 0.0001in all instances). We observed also a notable positive dependence (*P* < 0.0001) of the R_MZ_/R_DZ_ ratio on correlation between the repeated measurements (R_repeat_). The linear correlation between the R_MZ_/R_DZ_ and R_repeat_, ranged between 0.416 and 0.527, and in combination with the parallel elevation in correlation size between the twins, suggesting the increasing role of heritability with the increase in the longitudinal stability of the methylation site.

### 3.3. Identification of lsBINs associated with CWP in discovery set

We compared methylation levels of 723,029 lsBINs in 50 CWP discordant MZ twin pairs using the paired *t* test. Overall, 50,621 bins showed nominal significance for association (*P* < 0.05) which is close to the theoretically expected distribution (Fig. S1, supplementary material, available online at http://links.lww.com/PAIN/A395). However, the observed number of significant *P*-values is likely underestimated because of nonindependence of individuals, correlation between the methylation levels in twins, and nonindependence of bins, many of which are located very close to one another and display similar patterns of variation. Besides, the adjacent bins overlapped by 250 bps. All significantly associated bins were therefore tested in validation analysis.

### 3.4. Validation of association between selected lsBINs and CWP in replication set

A similar comparison of the methylation levels of all lsBINs in affected vs nonaffected twins was tested in our replication sample of 1608 individuals. The methylation levels were adjusted for age before analysis and then compared by *t* test. This resulted in 49,416 nominally significant differentially methylated lsBINs. We combined the significant results observed in both analyses implementing Fisher method (Fig. S2, supplementary material 1, available online at http://links.lww.com/PAIN/A395). In this method, nominally significant (*P* < 0.05) *P*-values obtained in the 2 independent samples were combined, restricting results to bins where the same direction of association between the methylation level and CWP- affection status was observed in both datasets. This generated 26,399 nominally significant signals with the same direction of the effect. We next checked whether the size of the association signals correlated with the R_repeat_. As seen in Figure S3 (supplementary material 1, available online at http://links.lww.com/PAIN/A395) there was a slight positive, but not statistically significant trend: the small surplus of the most significant associations (*P* < 0.01) was observed in R_repeat_ categories >0.30. The 24 most highly associated lsBINs by Fisher method are shown in Table 2.

**Table 2:**
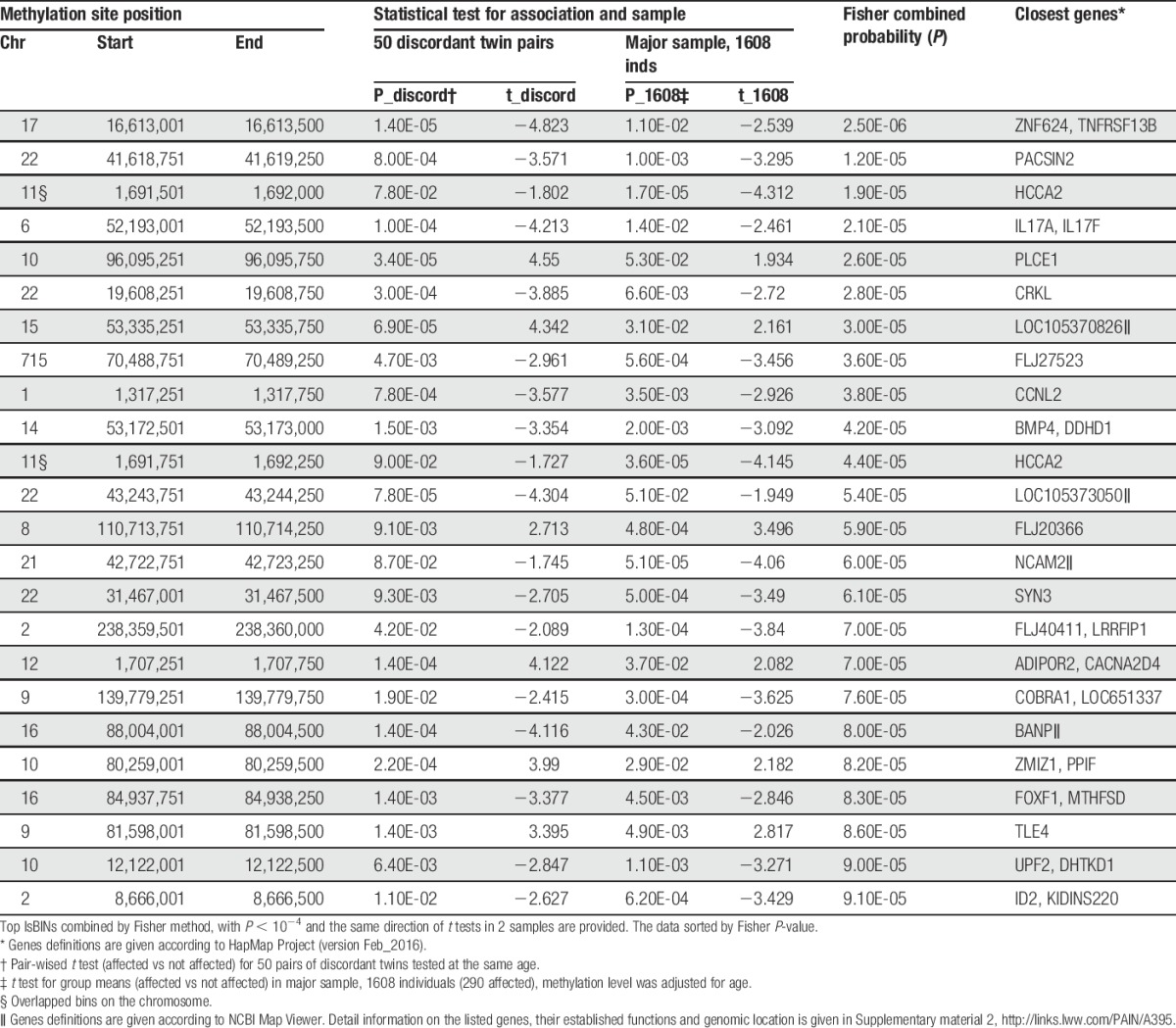
Summary of comparison of methylation levels between chronic widespread pain -affected and nonaffected individuals in 2 samples.

In total, we tested 723,029 bins. Our estimates of the effective number of independent tests (see M&M above) lay between 365,101 and 236,923, suggesting that the epigenome-wide significance level (accepting α = 0.05 for individual tests) is ≤1.36e-7. The lowest *P*-values for lsBINs associated with CWP were at the levels of “suggestive association” (https://www.biostars.org/p/141785/) ranging between 9.1e-5 and 2.5e-6, with no bin reaching epigenome-wide significance. However, it is also important that the direction of effect as established by the direction of the t statistic (positive or negative) was the same in both analyses with respect to >26,399 methylation sites, and in particular in all listed lsBINs (Table 2). That is, the bins showed elevated (or diminished) methylation levels in CWP-affected vs unaffected persons studied in both the discovery and replication datasets. As the 2 samples are independent, this finding adds confidence to the results. In addition, we estimated the proportion of methylation variation in top lsBINs attributable to CWP in the total sample and found it to be modest: it ranged between 0.31% (Chr#22, position starts 43,243,751) and 1.1% (Chr#8, position starts 110,713,751) by 1-way analysis of variance.

Next, we implemented multiple logistic regression tests with CWP as a dependent variable and methylation level of each bin as an independent variable and age, BMI and affection status of cotwin as covariates. Although the association of all listed covariates with CWP was statistically significant, the association of all the tested lsBINs also remained significant, with only a small change in the respective *P*-values (Table S1, supplementary material, available online at http://links.lww.com/PAIN/A395). Interestingly, smoking was significantly associated with CWP (*P* = 9.0E-05) in univariate analysis (Table 1) but was not retained in multiple logistic regression analysis for any of the tests (*P* consistently >0.05). We also tested for the independent effects of WBC subtype counts as covariates for each of the listed lsBINs and found no statistically significant result in any case.

Since the influence of the individual lsBINs on CWP may be nonindependent, we checked the combined effect of the1sBINs using multiple logistic regression. We simultaneously tested the association of top 4 lsBINs (because of limited sample size) with CWP diagnosis. The total sample of 1708 individuals was examined. The final regression model retained all 4 lsBINs included at the first stage of the analysis (Table 3), with virtually the same *P*-values as in their separate analysis (Table 1), suggesting the lsBINs are independent in their association with CWP. The contribution of other tested covariates (age, BMI, and cotwin affection status) also remained statistically significant and were independent from one another and from the effect of methylation levels, demonstrating that the epigenetic changes were not mediated through these known risk factors. Similar testing of 4 other lsBINs (listed among the top bins identified in multiple logistic regressions testing each bin separately, Table S1, http://links.lww.com/PAIN/A395) led us to the same conclusion, ie, association of each of the methylation sites with CWP is mostly independent from one another, and as well as independent from other tested covariates (not shown).

**Table 3:**
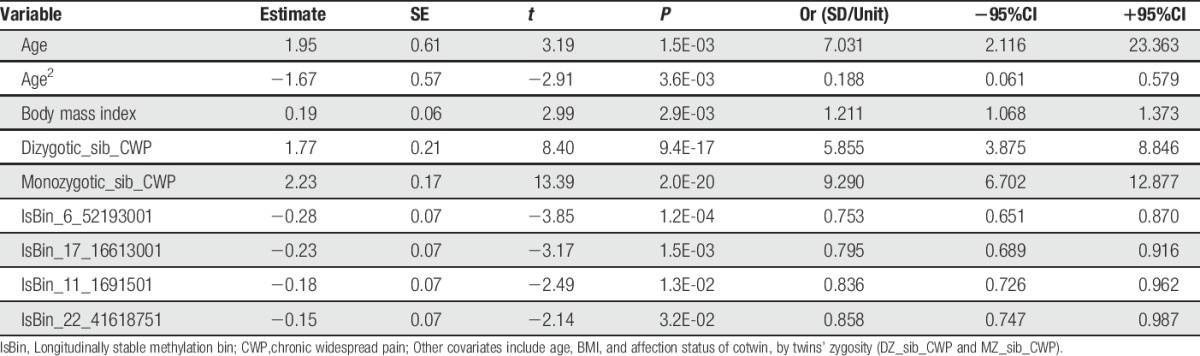
Multiple logistic regression analysis of chronic widespread pain association with 4 independent top longitudinally stable bins selected by smallest Fisher *P*-value (Table 1) in the total available sample (N = 1708 individuals).

### 3.5. Functional genomic annotations of bins most significantly associated with CWP

Using HapMap repository and GeneCards database we reviewed the genomic location and functional description of the most significantly associated bins. The list of the potentially functional genomic regions mapped to/or close to identified methylation signal(s) is provided in the right-hand column in Table 2. Their detailed description, according to chromosome and location is given in Supplementary material 2 (available online at http://links.lww.com/PAIN/A395). A preponderance of these genomic regions are involved in nervous system functions and inflammation. Thus, the top 3 bins included Chr17_16613001, annotated to overlap with the enhancer located near *ZNF624* gene known to be involved in transcriptional regulation and *TNFRSF13B* encoding tumour necrosis factor receptor; Chr22_41618751 mapped to protein kinase C and casein kinase substrate in neurons 2 (*PACSIN2*) gene. The protein encoded by this gene is involved in linking the actin cytoskeleton with vesicle formation by regulating tubulin polymerization; Chr11_1691501 located in vicinity of genes with no clearly interpretable or relevant to CWP function. Other bins of interest and biological relevance include: (1) Chr6_52193001 in vicinity of *IL17* gene. This gene encodes IL-17, a proinflammatory cytokine produced by activated T cells and is implicated in inflammatory arthritis and psoriasis; IL-17 blocking antibody is already in clinical use, confirming the IL-17 proinflammatory function and suggesting its possible involvement in pain symptoms. (2) Chr8_110713751 located within the syntabulin (*SYBU*) gene genomic region. Syntabulin is thought to mediate anterograde transport of mitochondria along neuronal processes. (3) Chr12_1707251 mapped close to the adiponectin receptor genes, *ADIPO-R1* and *-R2*, which serve as receptors for globular and full-length adiponectin which may provide the link with BMI predisposing to CWP. (4) Chr22_31467001 located in the genomic region that harbours synapsin III (*SYN3*) gene. This gene is a member of the synapsin gene family that encodes neuronal phosphoproteins, which associate with the cytoplasmic surface of synaptic vesicles.

### 3.6. Gene ontology and pathway analyses

The GO analysis tests for over-representation of GO terms in a given set of genes. For GO analysis we chose 1392 genes that were nominally significant (*P* < 0.01) in both the discovery and replication samples, and had a combined *P* < 0.001. We set up a threshold for GO *P*-values as *P* < 0.001.

Overall, 23 and 14 GO terms that passed the significance threshold were identified for BP and CC GO domains, respectively (Tables S2 & S3, supplementary material 2, available online at http://links.lww.com/PAIN/A395). The most significant GO term for BP domain was “positive regulation of GTPase activity” (*P* = 1.4e-11) represented by 78 genes, the top 10 including *TIAM1*, *RAPGEF1*, *DOCK9*, *SH3BP1*, *RASGEF1C*, *TBC1D5*, *RAP1GAP2*, *ASAP1*, *MCF2L*, and *AGAP1*. The most significant GO term for CC domain was “cell junction” (8.50e-11) represented by 141 genes, the top 10 including *GRIN2B*, *TIAM1*, *SHANK2*, *SH3PXD2A*, *SV2C*, *MAGI2*, *EGFR*, *PCDH9*, *GABRB1*, and *NUP214*. Notably, a number of the most significant GO terms are directly related to nervous system activity and development, eg, “neuron recognition”, “nervous system development”, “neuron projection”, “presynaptic membrane”, and others.

The importance of neurological components was further highlighted by the results of pathway analysis performed using Ingenuity Pathway Analysis (IPA) tool. For the same set of 1392 genes, the most highly significant canonical pathways were “Synaptic Long Term Depression” (*P* = 1.2e-8), “Axonal Guidance Signaling” (*P* = 3.4e-7), “CREB (cAMP response element-binding protein) Signaling in Neurons” (*P* = 4.3e-7), “Neuropathic Pain Signaling in Dorsal Horn Neurons” (*P* = 4.7e-7), and “Melatonin signalling” (*P* = 5e-7). The top diseases and biofunctions for “Physiological system development and function” in IPA analysis were “Behaviour” (*P*-values for different subcategories ranged from 3.1e-4 to 8.5e-7), “Reproductive System Development and Function” (*P* = 5.3e-3 to 6e-5) and “Nervous System Development and Function” (*P* = 1. e-2 to 2.3e-4). Finally, the top network built by the IPA was associated with “Nervous System Development and Function” category.

**Figure 2.**
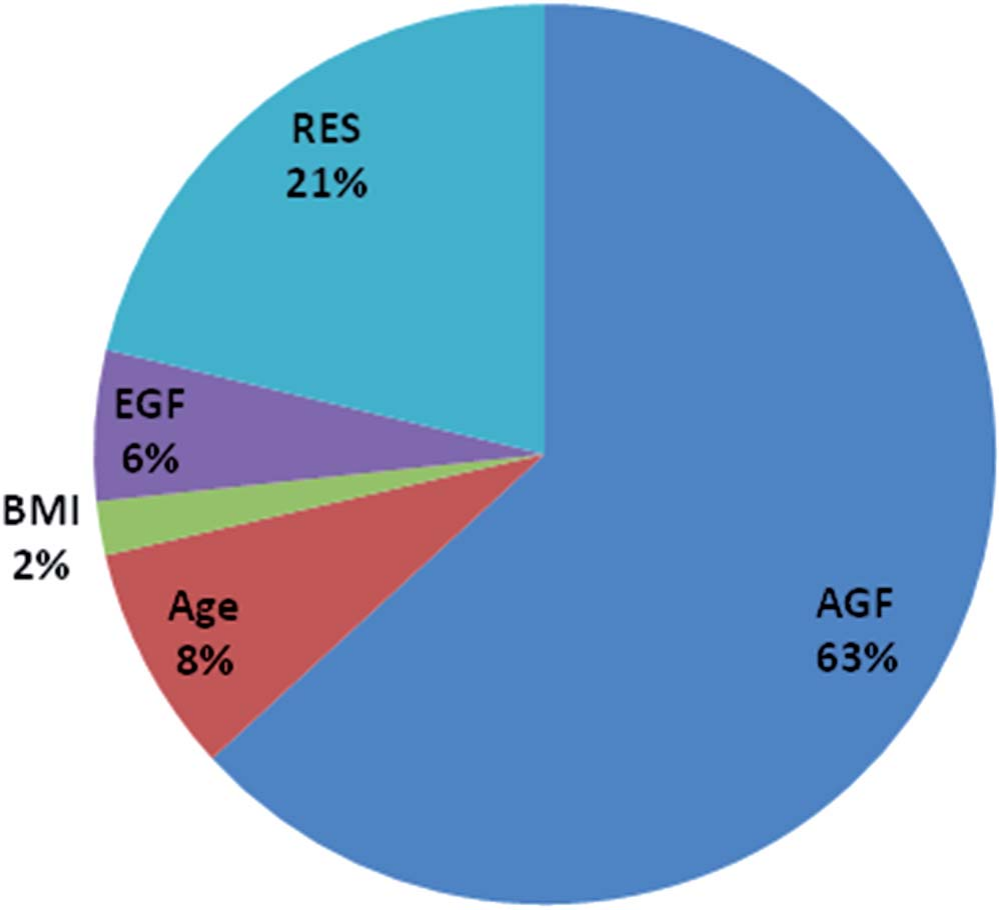
Summary of variance component analysis of chronic widespread pain -liability variation with covariates in total UK twins studied sample, 1708 individuals. AGF, additive genetic factors; EGF, epigenetic factors; RES, unexplained residual component of variation. The details of the implemented method given in Malkin et al.^34^

We were interested in the contribution of the DNA methylation signals relative to other risk factors to CWP. To this aim, we explored the relationship between genetic and epigenetic factors in CWP. Liability to CWP exhibited a significant heritability estimate of 0.636 ± 0.174. Permuting all possible combinations of the 24 most significant lsBINs (Table 3), we found the 4 top but also independent methylation sites. These bins, BMI and age were added into genetic model and the results are summarised in Figure 2. They confirm the importance of the genetic component for CWP (by likelihood ratio test, V_AD_ > 0; *P* = 0.015) and also showed that the 4 lsBINs have a significant association with CWP which was independent of genetic and other risk factors. The proportion of explained variance attributable to their effect varied between ∼1.0% (lsBIN_17_16613001) and ∼3.0% (lsBIN_6_52193001). The 4 bins together explain ∼6% of CWP-liability variance. These data show that the epigenetic influence at the top 4 loci are not acting on CWP through genetics, or BMI or age.

## 4. Discussion

We have conducted the first large-scale EWAS to identify methylation sites associated with CWP. Epigenetic factors, in particular DNA methylation, may be responsible for long lasting changes in gene expression, and may account for a substantial fraction of the “missing heritability”.^54^ On the other hand, an individual's epigenetic profile is dynamic and prone to environmental influence as well as genetic factors. Twins, particularly MZ twins, offer the ideal study subjects as MZ twin pairs are completely matched for genetic factors and age and often have very similar environmental influences, particularly in early development. We examined longitudinally stable methylation signals because CWP is a chronic, long term condition and it made biological sense to focus on the stable methylation differences in twins having vs not having CWP. After QC and testing of repeatedly measured methylation levels, we identified 723,029 methylation bins with statistically significant longitudinal correlation. These lsBINs had a significant additive genetic component (heritability), but represent only a minor fraction, about 11% of the total bins that passed the QC (n = 6,501,931). This is in agreement with other studies, eg,^9,56^ showing that the variation of most methylation sites is likely influenced by environmental factors. For example, van Dongen et al^56^ in their study of 411,169 methylation sites in 2603 individuals from twin families, found the average correlation in MZ twins (*r* = 0.20) was approximately twice as large as that in DZ twins (*r* = 0.09), and the average heritability estimate across the genome was ∼0.20, with very wide range between sites. The variation of lsBINs in this study, however, as attested by the significant twin correlations, is governed to a different extent by the genetic factors, with clear tendency to increase with the size of the correlation between the repeated measures. Smoking was significantly associated with CWP-affection status in our univariate analysis. It is also known to affect DNA methylation levels.^27,51^ However, in our multiple regression analyses it was not independently associated with pain, and also showed no significant correlation with our top lsBINs. It is possible the reason for this is that we examined longitudinally stable methylation sites, with a significant heritability component, and therefore less prone to environmental influences.

A comparison of the methylation levels between MZ-twins discordant for CWP revealed 50,621 nominally significant statistical differences. In the replication set, we identified 49,416 bins exhibiting nominal statistical significant associations with CWP. Combining the results resulted in 40,916 nominally significant results (*P* < 0.05), of which 24 had *P* < 10^−4^. However, they were obtained using different approaches in 2 virtually independent samples, and not only showed significant differences between the groups of affected and nonaffected individuals but also differences that were in the same direction.

A GO enrichment analysis identified a number of terms enriched among high-ranking methylation sites that were in agreement in the discovery and replication sets. The top GO category in BP domain was GO:0043547 “positive regulation of GTPase activity”. Importantly, over the past decade it has become obvious that GTPase activity is crucially important in many aspects of neuronal development, such as neurite outgrowth, axon pathfinding, neuronal differentiation, dendritic spine formation, and maintenance. Mutations in genes involved in GTPase activity are known to be associated with neurological diseases in humans.^17^ Our top differentially methylated loci contributing to this BP have been found to be critically involved in neurogenesis. For instance, *TIAM1* gene has been shown to be involved in neurons morphology and axon formation^25,53^ as well as neurite outgrowth.^50^ *RAPGEF1* gene is essential for such aspects of cortex development as radial glial attachment and neuronal migration^59^ as well as in the establishment of a polarized morphology of developing neurons.^49^ *DOCK9* gene is important for the growth of dendrites in hippocampal neurons.^26^ *MCF2L* gene may be involved into IL1RAPL1-dependent formation and stabilization of glutamatergic synapses of cortical neurons.^19^ *AGAP1* was found to affect dendritic spine morphology and endosomal traffic in neurones.^2^ This gene also mediates dopamine release through an interaction with M5 muscarinic acetylcholine receptor.^6^

These findings suggest that the differential methylation of genes is important for the development and maintenance of neurons and the nervous system and may be an important factor in the development of CWP. This conclusion is further supported by the finding that other most significant GO terms for BP and CC domains were related to the neurological system and sensory functions, such as “neuron recognition,” “neuron projection,” “neuronal cell body,” and variety of synapse-related features.

Also, the top network built by the IPA was associated with the “Nervous System Development and Function” category. Moreover, some of the genes contributing to these GO terms and pathways have previously been implicated in various pain phenotypes. For instance, the *GRIN2B* gene was found to be important in neuropathic pain^60^ and is essential for neuroplasticity.^11^ CABRB1 gene associated with pain related movement limitations.^38^

Remarkably, the most significant lsBINs associated with CWP are located within or around highly plausible candidate genes (according to analysis of corresponding genomic region annotations) with respect to neurological and other relevant aspects of CWP pathogenesis. The examples include *PACSIN2, SYBU,* and *SYN3* genes among top association signals, all related to neuronal activity. Two others, tumour necrosis factor receptor and *IL17*, are related to inflammation. One of them, *IL17*, encodes respective proinflammatory cytokine which was implicated in pelvic pain^42^ and experimental neuropathic pain.^24^ Finally, *ADIPOR1* and *ADIPOR2* genes encoding receptors for adiponectin, a protein expressed in many tissues, (including central nervous system) and involved in regulation of BMI and inflammatory pain regulation.^20^ These genes also may be a part of a functional bridge connecting BMI^44^ and specifically fat mass to CWP, a linkage which we have already explored.^33^

Thus, the results of the association analysis and GO/pathway analysis are complementary in terms of pointing to a neurological component in CWP development and manifestation. The summary analysis of the various potential risk factors to liability to CWP, tested in this study suggests that although the additive genetic component remains the leading factor (∼63% of the total variance), the contribution of epigenetic factors is also sizable, and explains at least 6% of the variation in CWP.

The study has several limitations. The twins studied are community dwelling volunteers and therefore are not the same as hospital patients, in terms of severity and accuracy of case assignment which was performed by validated questionnaire. However, the benefit of this approach is the availability of a large sample which can address major social and medical problems. There is no standardised definition for the diagnosis of CWP; we used a validated questionnaire, which was the best tool available for epidemiological study at the time,^61^ and have continued with this method for the generation of longitudinal data to allow accurate comparison case control status over time. As CWP shares genetic predisposition with other types of pain,^34,58^ the results of any analysis may depend on the phenotypic composition of the sample. There are also statistical issues related to the multiple testing problem that appears at different stages of the analysis, including computation of the large number of correlations to identify lsBINs and numerous association tests between them and CWP. Taken together this suggests that large consortia having standardised CWP definitions will be needed to define precisely the methylation loci predisposing to CWP.

Note also that the analysis was based on whole blood, which is not the optimal tissue to study in CWP. Although human tissue from dorsal root ganglia or brain are becoming available [http://www.iiam.org/humanTissueForResearch.php; Ref. 12] large collections of this type of tissue from CWP patients are unlikely to be gathered. It is important to note, however, that there is a correlation between brain methylation and that seen in whole blood,^12^ so we believe that our results likely reflect methylation patterns in nervous system tissue to some extent. This is especially highlighted by the GO analysis for CC domain, as the molecules whose genes contributed to the most significant GO terms, operate within neurons and their compartments, such as synapses. Finally, variations in blood cell composition may affect the results of the methylation analysis. However, in the subsample having WBC composition information (n = 441), the pattern of associations between methylation and CWP for the 10 top-ranked CWP-associated signals remained unchanged with inclusion of the WBC composition in the regression model (data not shown). Thus, blood cell composition did not influence our results.

In conclusion, this large study of DNA methylation in twins provides evidence of epigenetic change in DNA accounting for 6% of the liability to CWP. Functional analysis of the genes in close vicinity to differentially methylated regions showed highly significant enrichment for neurological pathways. There is a clear need for further examination of the potential role of DNA methylation, and the use of other omics, to understand better the biological mechanisms leading to CWP. The pathways identified provide an important starting point for the identification of novel targets for therapeutic intervention.

## Conflict of interest statement

All the authors of the present manuscript declare that they have no conflict of interest.

I.M. and M.B.F. authors contributed equally to the work

Supported by Arthritis Research UK (grant number 20682) and by the Israel Science Foundation (grant number 1018/13).

Further support for this work was obtained from the European Research Council (ERC 250157) and the EU-FP7 project EpiTrain (316758). TwinsUK NIHR BRC Bioresource: is funded by the Wellcome Trust; the European Community's Seventh Framework Programme (FP7/2007-2013); and the National Institute for Health Research (NIHR) BioResource, Clinical Research Facility and Biomedical Research Centre based at Guy's and St Thomas' NHS Foundation Trust and King's College London.

This study received ethics approval from the St. Thomas' Hospital Research Ethics Committee.

## Appendix A Supplemental Digital Content

Supplemental Digital Content associated with this article can be found online at http://links.lww.com/PAIN/A395.
